# Analysis of urban heat island characteristics and mitigation strategies for eight arid and semi-arid gulf region cities

**DOI:** 10.1007/s12665-021-09540-7

**Published:** 2021-03-22

**Authors:** Ammar Abulibdeh

**Affiliations:** Department of Humanities, College of Arts and Sciences, Qatar University, Doha, Qatar

**Keywords:** Urban heat island, Land surface temperature, Land cover, Arid and semi-arid region, Mitigation policies

## Abstract

The aim of the study is, therefore, to analyze the formation of the UHIs in eight different cities in arid and semi-arid regions. The analysis is based on land cover (LC) classification (urban, green, and bare areas). The study found that bare areas had the highest mean LST values compared to the urban and green areas. The results show that the difference in temperatures between the bare areas and the urban areas ranges between 1 and 2 °C, between the bare areas and green areas ranges between 1 and 7 °C, and between the urban areas and green areas ranges between 1 and 5 °C. Furthermore, the LST values varied for each of the LULC categories, and hence some areas in the three categories had lower or higher LST values than in other categories. Hence, one category may not always have the highest LST value compared to other categories. The outcomes of this study may, therefore, have critical implications for urban planners who seek to mitigate UHI effects in arid and semi-arid urban areas.

## Introduction

Rapid urban sprawl typically results in the conversion of open spaces, such as natural land cover consisting of soil and vegetation, into artificial surfaces, which consist of concrete, asphalt, and other impervious surfaces. This process inevitably results in changes in the absorption and reflection of solar radiation and the balance of surface energy. It also results in contrast changes to the urban-surroundings/urban–rural environment, in terms of surface radiance and air temperature. This difference in ambient air temperature between the urban-surroundings/urban–rural areas results in a phenomenon known as Urban Heat Islands (UHI) (Aflaki et al. 2017; Mohajerani et al. 2017; Zhao et al. 2016; Santamouris 2015). This variation in temperature is what constitutes the UHI effect. Various studies have found that UHIs can increase the air temperature in urban areas between 2 and 15 °C (Aflaki et al. 2017). In arid and semi-arid regions, the temperature in urban area centers can increase in the range of 2–4 °C when compared to their surroundings (Wong et al. 2016; Martin et al. 2014). The spatial patterns of UHIs in urban areas reflect the different natures of the various components and factors that affect them. One main reason that causes this phenomenon is human activity, which creates dense, built-up areas that produce excess heat energy. This energy is captured by UHIs during the day and is diffused at night (Oke 1982).

The thermal balance and the intensity of an UHI is a function of different elements, including weather and climate conditions (i.e., rainfall availability, moisture), urbanization, population dynamics, the size and the density of an urban area (horizontal and vertical urban expansion), morphology (topography and sky view factors), urban design (spacing between buildings and location of public places), water bodies and sea reclamation, land use/land cover (LULC) characteristics, the ratio of green areas, anthropogenic heat production, and the types of materials used in construction (Qaida et al. 2016; Grover and Singh 2015; O’Malleya et al. 2015). These factors will affect the intensity and spatial patterns of UHIs. On the other hand, UHIs will affect surface temperatures, evaporation rates, energy consumption rates, degrees of solar radiation absorption, impair water quality transmission from the heat to the soil, elevate greenhouse gases and emissions of air pollutants, the albedo of the area, heat storage capacity, and ultimately will jeopardize human health and comfort. Additionally, UHIs can affect wind turbulence, which can directly alter the near-surface atmospheric environments over urban areas (Estoque et al. 2017; Zhou et al. 2016).

The characteristics of manmade materials are another factor that influence UHIs. These materials, which are used in transforming the natural surface to urban built-up elements, can store shortwave radiation, release latent and sensible heat through evaporation and convection processes, emit infrared radiation, block prevailing winds, and store heat (Boehme et al. 2015; O’Malleya et al. 2015; Dimoudi et al. 2014). The optical and thermal features of the material (i.e., density, thermal capacitance, short-wave radiation reflectivity, conductivity, and emissivity) have significant impacts on the distribution of the ambient temperature in urban areas (Coseo and Larsen 2014). On the other hand, the surrounding areas are usually composed of natural surfaces (i.e., plants and soils) that release water vapor into the air and keep the area cool.

A significant number of studies have thoroughly investigated and analyzed the UHI phenomenon, which has created feedback for researchers and policymakers (Meng et al. 2018; Gu and Li 2018; Jun et al. 2017; Yang and Lin 2016; Bokaiea et al. 2016; Wang et al. 2016). The relationship between urban features and UHIs can be summarized in four key points, as suggested by the literature (Gago et al. 2013; Jun et al. 2017): (1) green areas, pavement, and buildings are the most significant elements on temperature variations at the local level; (2) the distribution of buildings and urban structures in urban areas influences the development of the UHI phenomenon; (3) the presence of tall buildings and narrow streets increases the effects of UHIs as they capture warm air and decrease airflow; and (4) water and green spaces are significant components that can mitigate the effects of UHIs.

In the literature, LST has been identified as one of the most significant factors that can influence the UHI phenomenon (Song et al. 2014; Zhou et al. 2011). LST information has been widely used in UHI research and is usually obtained from remote sensing data, which is a unique source of information for determining UHIs (Tran et al. 2017; Di Leo et al. 2016; Feyisa et al. 2014). These studies used remote sensing images to retrieve land-use information, which then classified LULC into the following categories: built-up areas, green areas (i.e., parks, forests, vegetation), water, or recreational land (Heusinkveld et al. 2014; Choi et al. 2014; Emmanuel and Loconsole 2015; Fan et al. 2015; Fadda et al. 2019). Many studies emphasized the relationship between LST and LULC and its consequences on UHIs. These studies concluded that LST is directly correlated with built-up areas and has an inverse relationship with green areas. The examination of LST data based on LULC changes is significant enough to initiate policies that can mitigate the effects of UHIs, as well as adopt new land-use planning and urban design strategies and policies for arid and semi-arid urban areas. Several studies used remote sensing images for the investigation, derivation, and monitoring of LST and UHI effect (Duan et al. 2017; Berger et al. 2017; Estoque et al. 2017; Jalan and Sharma 2017; Makido et al. 2016). These images are inimitable dataset, which make it possible of attaining frequent wall-to-wall coverage of a coveted context. Furthermore, satellite remote sensing provides a straightforward and consistent method of measuring LST over extended regions and hence it is considered the most effective method in studying the UHI effect and in measuring the LST at regional and global scales (Duan et al. 2017). The thermal bands of these images facilitate the process of obtaining and deriving the UHI intensity (Berger et al. 2017; Jalan and Sharma 2017; Pan 2016). These images can be used to derive the LULC and LST and subsequently UHI intensity. The utilization of thermal bands of Landsat TM/ETM/OLI in several studies can be attributed to many factors such as availability, world-wide coverage with a good spatial resolution, long-term temporal coverage, and the presence of thermal and thematic spectral bands that are important to study the UHI phenomenon (Deilami et al. 2018). Satellite thermal infrared (TIRS) measurements are widely used to retrieve LST with relatively high accuracy and high spatial resolution. On the other hand, some limitations associated with utilizing these images include a long time of the satellites revisit to the same area (16 days), the large image size, and their inability to penetrate clouds. In addition, the spatial resolution of thermal and thematic bands may not be suitable for detailed LST/UHI investigation. Furthermore, different elements may affect the precise measurement of emissivity (such as noise of sensor, atmospheric effect, aerosols) and hence the measurement of LST (Krehbiel and Henebry 2016; Jiménez-Muñoz and Sobrino 2006). These limitations make these images not suitable for monitoring changes in LST within the day or a short period as well as the need to process multiple images to mosaic them when focusing on national or regional scales, which increase the processing time significantly (Deilami et al. 2018). Nevertheless, in arid and semi-arid areas (such as our study area), the sky is clear from the clouds most of the time during the year particularly in the summer season, which overcome the limitation of using satellite images in studying the UHI effect.

Different methodologies and techniques were used to investigate the UHIs phenomena and to classify the LULC. Some studies used model simulation (Litardo et al. 2020; Giridharan and Emmanuel 2018; Li et al. 2018; Kleerekoper et al. 2017). For example, this method was used for studying the effect of urban compactness and different urban layouts under temperate climate conditions on UHI. Other studies used the model simulation method to investigate the influence of different greenery and thermo-physic parameters and different types of pavements (i.e. leaf evapotranspiration rate, albedo), or specific urban layouts on the formation and intensity of UHI phenomenon. These models are used to address what if scenarios, but not the real parameters of the problem.

Other studies used statistical analysis of temperature records from observational stations in investigating UHI phenomenon. Some studies used the ordinary least squares (OLS) regression is associated with Pearson’s correlation (Henits et al. 2017; Estoque et al. 2017; Chen et al. 2017; Haashemi et al. 2016; Fan et al. 2015; Zhao et al. 2014). This model is used to examine the UHI effect for a large area. However, other studies (Deilami and Kamruzzaman 2017; Deilami et al. 2016) find that UHI effect varies significantly over space (i.e. context sensitive) and hence this phenomenon should be modelled locally. Therefore, some studies used geographical weighted regression (GWR) models to investigate the UHI (Deilami et al. 2016). The GWR was found to have better explanatory power than OLS model. Some studies used comparative analysis for investigating and modeling this phenomenon (Guo et al. 2020; Jalan and Sharma 2017; Pal and Ziaul 2017; Singh et al. 2017; Cui et al. 2016). This method is based on investigating the UHI effects and the classification of LULC. To provide comprehensive results, this method was used in some studies with other statistical methods such as the OLS.

Several studies have investigated the relationship between LST and the distribution and spatial patterns of impervious urban surfaces and green areas, but few have considered cities in arid and semi-arid regions. Many of these studies focused on individual cities, however, a study that considers the Gulf region is lacking, and comparative monitoring of UHIs in various cities is rather limited. Therefore, this study investigates the relationship between LST and the abundance and spatial pattern of impervious urban surfaces, green areas, and bare areas in multiple cities in the Gulf region. This study differs from others in several ways. First, no other studies have empirically analyzed the impacts of LC changes on the development of UHIs in eight main cities in the Gulf region. Second, this study assesses the spatiotemporal variations of the UHIs over eight major cities. Finally, this study contributes to the literature by examining the interactions between land cover and LST over time in arid and semi-arid regions.

This study aims to investigate the impacts of LC on LST, and hence the development of UHIs as well as the mitigation processes in eight different urban metropolitan cities in arid and semi-arid regions using multi-spectral and multi-temporal satellite data. The study investigates how urbanization and population growth affect the thermal behavior of UHIs. The LSTs and the normalized difference in vegetation indices (NDVI) were retrieved from remote sensing images. In this study, spatial and temporal LST data were extracted in three phases (e.g., in 1990, 2000, and 2018) and were analyzed for spatial distribution changes in temperature and LULC, using Landsat images. A quantitative approach was used to investigate the relationships between LULC, NDVI, and LST. High spatial and temporal resolution Landsat-5 Thematic Mapper (TM) and Landsat 8 OLI/TIRS data were used to analyze, map, and assess spatial and temporal variations in UHIs and determine their relationship with urban surface characteristics. For each city, LST for green areas, urban areas, and bare areas were analyzed. This study mainly focused on the summer period with the following objective: to assess the spatial distribution of LST and its correlation with LULC, as well as the role of LULC in the development of atmospheric UHI and the resulting variations in the microclimate within the canopy layer.

## Study area

**Fig. 1:**
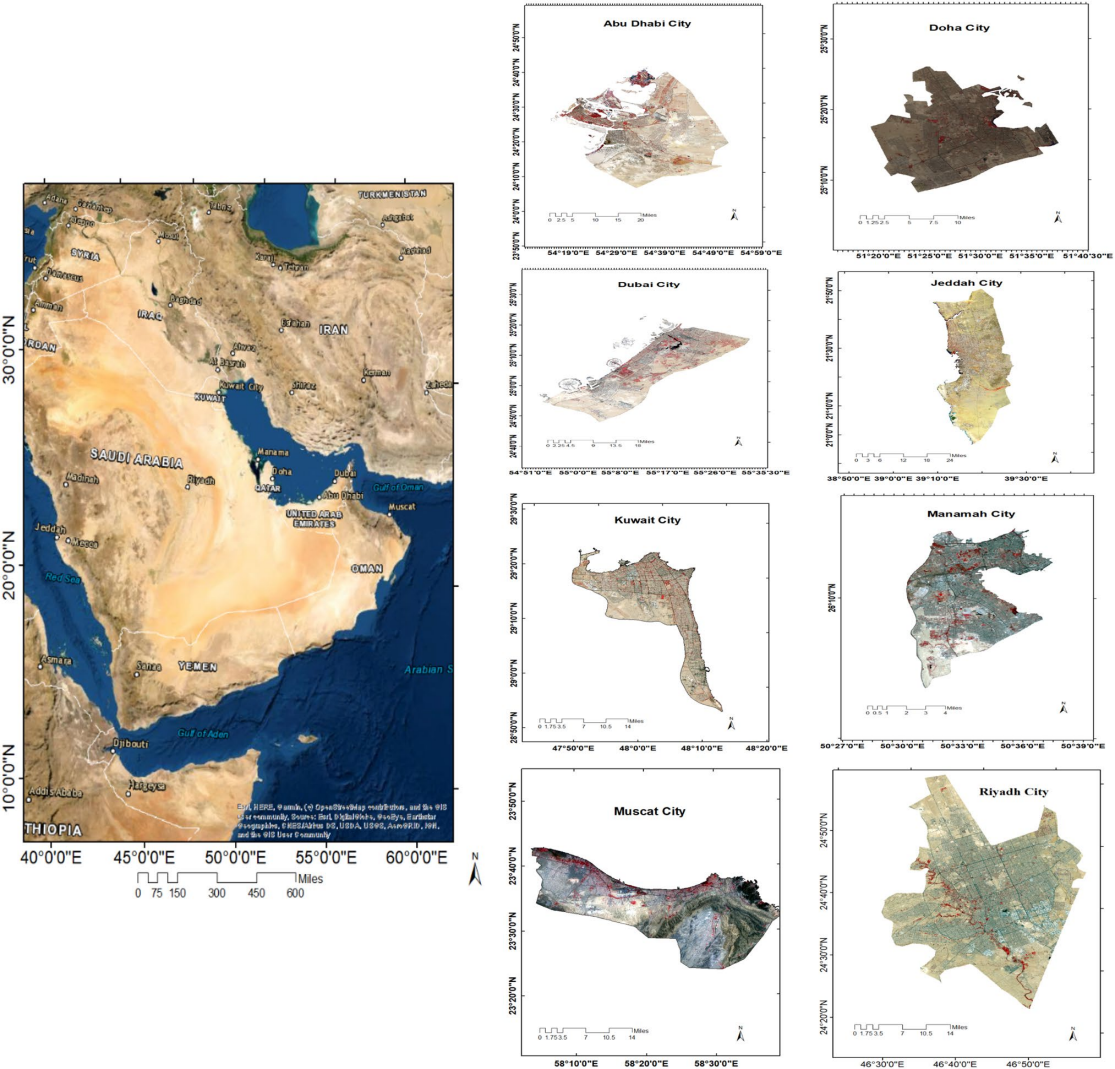
Study area

The cities observed in this study were: Doha in Qatar, Abu Dhabi and Dubai in the United Arab Emirates (UAE), Riyadh and Jeddah in the Kingdom of Saudi Arabia, Muscat in the Sultanate of Oman, Kuwait City in Kuwait, and Manamah in the Kingdom of Bahrain (see Fig. 1). The eight cities are primacy cities in the Gulf Region and the core of their countries in population and economic growth. In addition, these cities have been among the most rapidly growing cities in the world over the last few decades, and serve as hubs for regional development.

### Climate characteristics

**Fig. 2:**
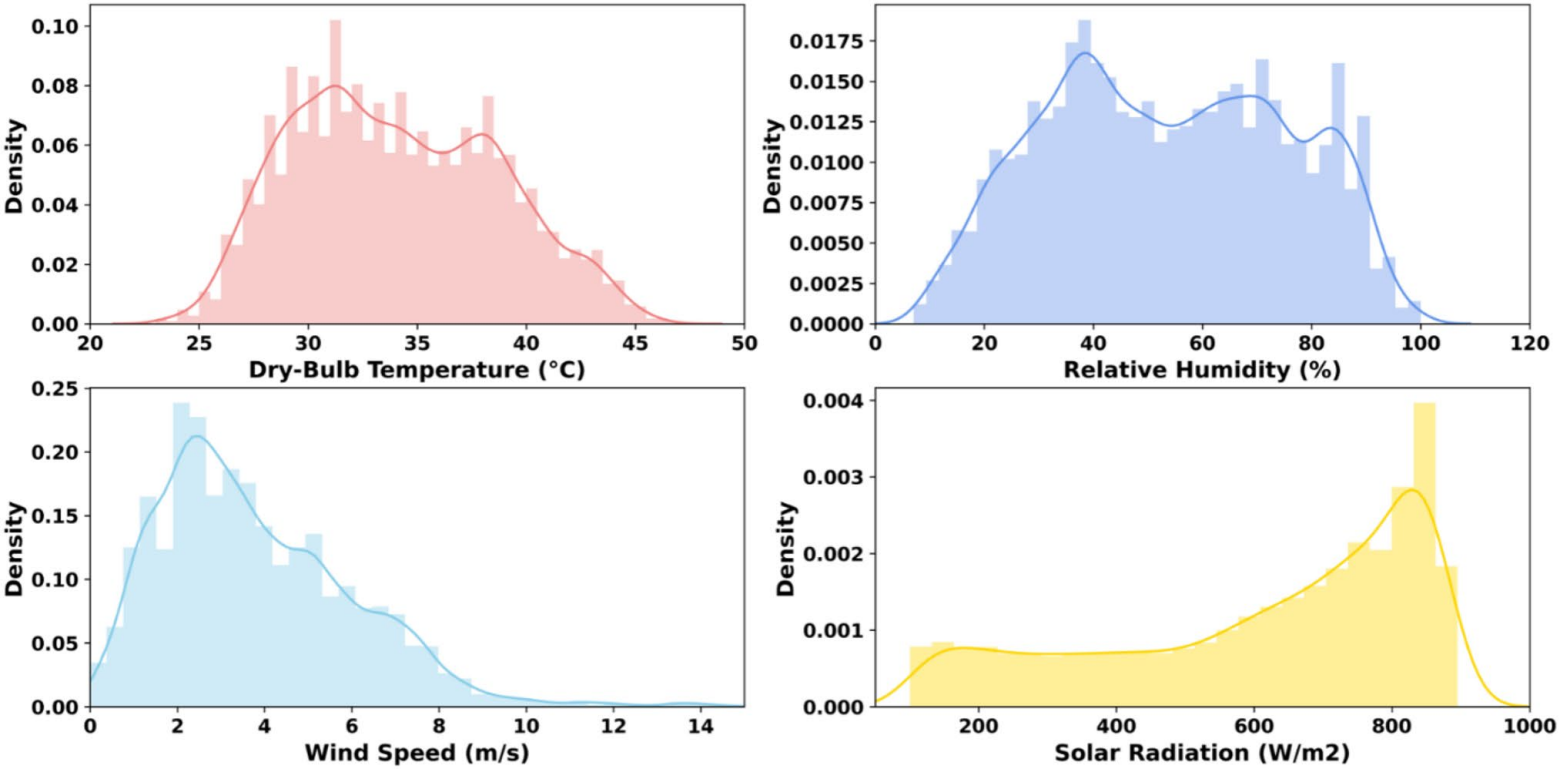
Normalized distribution of the climate characteristics for Doha, Qatar during the summer (for June, July, and August)

According to Koppen-Geiger climate classification, the Gulf region, including the eight cities, is classified as Tropical and Subtropical Desert Climate (BWh). The Region is characterized by having two seasons, summer and winter. The summer season extends between April and October and it is scorching, where the average temperature ranges between 32 and 38 °C (see Table 1), and the temperature can rise to 50 °C in the continental cities such as Riyadh with low relative humidity during June or July. The precipitation is minimal during the summer season. Simultaneously, the temperature in some coastal cities can rise to more than 40 °C such as in Kuwait City with relative humidity up to 90%. On the other hand, the winter season extends between November and March and is characterized by moderate temperature as the average temperature in the coldest months ranges between 13 and 23 °C and could rise to 26 °C in some coastal areas. The relative humidity is low during the winter season. The precipitation is sparse and ranges between 43 and 160 mm. The precipitation in the winter is mainly due to the cold northern winds from the Mediterranean region meeting the weather depression from the Indian Ocean. Figure 2 shows an example of the normalized distribution of the climate characteristics for Doha City during the summer season.

**Table 1:** Koppen-Geiger climate classification for the study area

City	Climate classification	Climate characteristics
Doha	BWh*	Average annual temperature = 27.2 °C (Summer 35.6 °C, winter 17.2 °C), average annual precipitation = 81.3 mm, the average annual percentage of humidity is: 58.9% (weatherbase, 2020)
Kuwait	BWh	Average annual temperature = 26.1 °C (Summer 37.8 °C, winter 12.8 °C), average annual precipitation = varies from 75 to 150 mm, the average annual percentage of humidity is: 60.0%
Muscat	BWh	Average annual temperature = 28.9 °C (Summer 35.6 °C, winter 21.7 °C), average annual precipitation = 149.9 mm, the average annual percentage of humidity is: 67.0%
Abu Dhabi	BWh	Average annual temperature = 27.2 °C (Summer 35.0 °C, winter 18.3 °C), average annual precipitation = 129.5 mm, the average annual percentage of humidity is: 75.4%
Dubai	BWh	Average annual temperature = 27.2 °C (Summer 35.0 °C, winter 18.9 °C), average annual precipitation = 160 mm, the average annual percentage of humidity is: 57.0%
Manamah	BWh	Average annual temperature = 26.7 °C (Summer 34.4 °C, winter 14.4 °C), average annual precipitation = 71 mm, the average annual percentage of humidity is: 73.0%
Riyadh	BWh	Average annual temperature = 26.1 °C (Summer 36.1 °C, winter 16.7 °C), average annual precipitation = 109.2 mm, the average annual percentage of humidity is: 29.0%
Jeddah	BWh	Average annual temperature = 28.3 °C (Summer 32.2 °C, winter 23.3 °C), average annual precipitation = 43.2 mm, the average annual percentage of humidity is: 62.8%

*BWh: Tropical and Subtropical Desert Climate

### Morphology characteristics

During the last four decades, the eight cities have been developed in a desert environment and transformed from small cities to sprawling metropolitan areas due to the discovery of large gas and oil reserves. These cities have almost similar urban morphology, albeit of some differences due to economic and population growth. Seven cities are coastal cities and only Riyadh city is a continental city. Muscat is the capital and the largest city in the Sultanate of Oman and is situated along the Oman Sea in the northeast of the country. The urban expansion of Muscat is mainly in a horizontal direction along the coastal strip (Abulibdeh et al. 2019a). Consequently, population density has decreased despite the rapid population growth. Between 1990 and 2017, Muscat witnessed an average annual urban expansion rate of 30%, while the green areas increased by an average annual expansion rate of only 0.024% (Abulibdeh et al. 2019a). Abu Dhabi is the capital of the United Arab Emirates and the largest city. It is situated on the east cost of the Arabian Gulf. The City witnessed a significant transformation from a fishing village to a contemporary city (Abulibdeh 2020; Abulibdeh and Zaidan, 2018; Hawas et al. 2016). Dubai is the second-largest city in the UAE and is located at the west coast of the Arabian Gulf. The city was transformed into one of the main commercial and real state hub in the region (Zaidan and Abulibdeh 2020). Dubai has witnessed a rapid growth of its population, expanding from a small town of 20 thousand residents, seven decades ago, to almost 3 million residents in 2020. Furthermore, urbanization in the city extended 400 times to include 1309 high-rise buildings within 3885 sq. km of land. Between 1990 and 2017, Dubai expanded on an average annual rate of 17%, while the green area expanded on an average annual rate of 0.18% (Abulibdeh et al. 2019a). Dubai has formed an image for itself as the most audacious of the Gulf cities primarily due to its numerous megaprojects. Some of these megaprojects include the Internet City, which was one of the initial large-scale projects to be executed, with the goal of transforming the urban area into a hotspot for Internet and Communication Technology firms. Other megaprojects are Media City, Dubai Marina, Palm Jumeirah, Burj Khalifa and Dubai Mall, and Dubai World Central is an airport city that will also accommodate the pavilions for the 2020 Expo (postponed to 2021 due to COVID 19 crisis) (Zaidan and Abulibdeh 2020).

Riyadh is the capital city of Saudi Arabia and the largest with a population of around 6.5 million. The City is situated in the middle of the country, away from the two costs of the country. The population growth rate is about 4% and the city is expanding horizontally. It is estimated that the housing units in the city are around 1.2 million (Aina et al. 2017). Jeddah city is the second-largest city in Saudi Arabia. It is situated on the Red Sea in the west coast of the country. Land use and land cover in this city witnesses a rapid change during the past four decades, with residential, industrial, commercial, public spaces, and informal settlements are the significant land use classes in the city. The population number exceeds three million people (Miky 2019). Manamah is the capital city of the Kingdom of Bahrain and most dense. It is situated in the northeast of Bahrain on the eastern coast of the Arabian Gulf with the most social, cultural, and economic activities. Kuwait City is the capital and the largest city of Kuwait, with a population of about 4 million. The majority of the population is urbanized. It is situated in the southern part of the country on the Arabian Gulf.

**Fig. 3:**
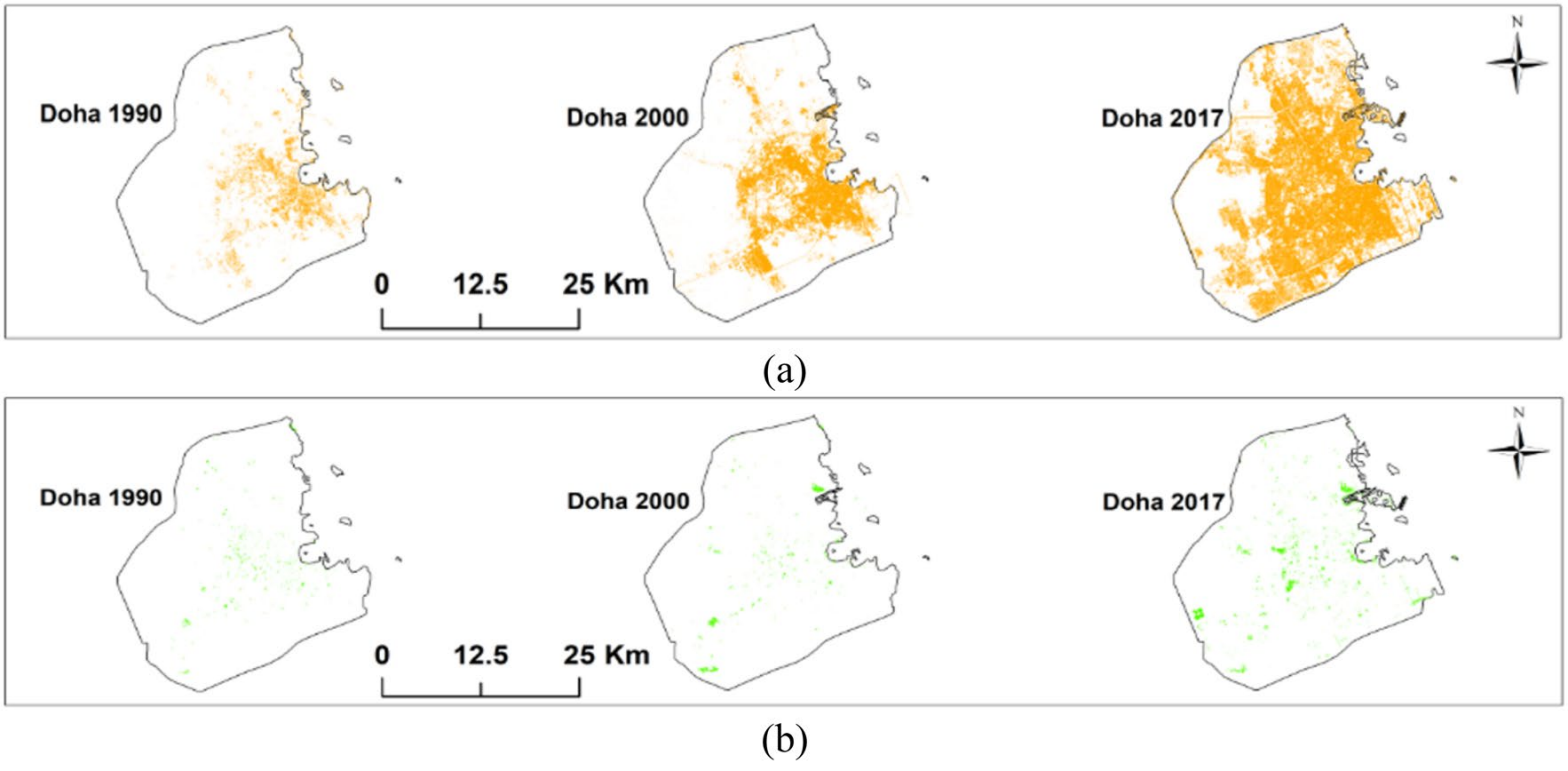
**a** The distribution of the urban area in Doha city between 1990 and 2017, **b** The distribution of the green area in Doha city between 1990 and 2017 (Abulibdeh et al. 2019a)

**Fig. 4:**
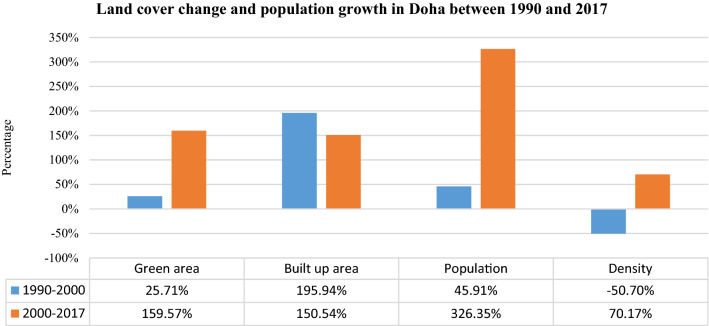
Land cover change and population growth in Doha between 1990 and 2017 (Abulibdeh et al. 2019a)

Doha city is the capital and the most populous city in the State of Qatar. It is located at the east coast of the Arabian Gulf in the east of the country. The City experienced significant urban growth during the past three decades due to oil and gas resources (Zaidan and Abulibdeh 2018). Between 1990 and 2017 period, the urban area expanded by 641.45%; however, the expansion of the urban area was at a higher rate between 1990 and 2000 (19.65%) than between 2000 and 2017 (8.85%) as shown in Fig. 3a (Abulibdeh et al. 2019a). On the other hand, the green areas in the city increased as well, however, it occupied only 1.28% of the city as of the year 2017 as shown in Fig. 3b (Abulibdeh et al. 2019a). The average annual expansion of these areas was 2.57% between 1990 and 2000 period, and at an average rate of 9.38% per year after 2000. Figure 4 shows that the city had experienced significant urban and population growth and consequently population density had an increase in the urban areas.

In the past few years, few studies have investigated the UHI phenomenon within the arid and semi-arid Gulf region (see Table 2). These studies analyzed the UHI on a microscale level utilizing different techniques and different data sources. The data consists of air temperature, LULC variables, and thermal bands of radiometric instruments flown on satellites. Some of these studies used different statistical approaches such as Random Forest, Regression Tree Analysis, and Ordinary Least Squares to explain near-surface air temperatures. Other studies used image processing techniques to investigate the formation of UHI phenomenon and the associated LST. Some of these studies found that UHIs migrate throughout the day (Makido et al. 2016), while others found that the UHI magnitude in the summer season is higher than in the winter (Elghonaimy and Mohammed 2019; Nassar et al. 2016; Charabi and Bakhit 2011). Furthermore, the variation in temperature between the urban and non-urban areas varies in the range of 4–7 °C (Miky 2019), 3–5 °C (Al-Blooshi et al. 2020), and 2–5 °C (Radhi et al. 2013).

**Table 2:** Different studies investigating UHI effects in the study area

Study	Place	Aim of the study	Data	Methodology	Results
Makido et al. (2016)	Doha, Qatar	To examine the spatial and temporal variation of near-surface temperature and the impact of urban form on temperature moderation	Air temperatures, diurnal cycles, land cover variables	Ordinary Least Squares (OLS), Regression Tree Analysis (RTA), and Random Forest (RF)	The UHI is a dynamic phenomenon during the daytime, where it migrate based on the features of the landscape that gain and release thermal capacity at different rates during the day. In addition, the Random Forest model is found to be more accurately predict the LST than the other methods
Elghonaimy and Mohammed, (2019)	Manama, Bahrain	To investigate the formation of UHI considering the influence of the limitations of public space and urban density. Furthermore, to study the impact of green roof systems in mitigating the UHI phenomenon	Remote Sensing Data and Thematic maps collected and extracted by the Sentinel 2B MSI satellite from Landsat 8	Analytical strategy of forest fragmentation and ArcGIS 10.5	The UHI in the winter is less than in the summer. This is due to the microclimate conditions of the city as well as to that the solid area is larger or equal to the void area, which have negative consequences in the ventilation of the UHI
Miky (2019)	Jeddah, Saudi Arabia	To analyze surface urban heat island (SUHI) over Jeddah city	Landsat 8′s Thermal Infrared Sensor (TIRS) night vision images and Spot5 data, land cover pattern	Improved mono-window algorithm	The variation in temperature between urban and non-urban areas ranges between 4 and 7 °C. In addition, the SUHIs appeared as small boundaries in the south area of the city, as a result of the land use patterns
Aina et al. (2017)	Riyadh, Saudi Arabia	To investigate the spatial and temporal changes in land surface temperature (LST) influenced by land use/land cover types	Multi-temporal Landsat images of the study area, 1985, 1995, 2002 and 2015 Land use/land cover types	One-way analysis of variance (ANOVA) Calculating UHI index for various LU/LC types using SUHI	The highest temperature was found in the industrial areas, particularly along the transportation network, while vegetation areas have the lowest. Furthermore, the results shows a trend of rising temperature in all the land use types
Al-Blooshi et al. (2020)	Al-Ain City, UAE	To study the impact of desert urbanization on urban heat islands effect	Analyses of multi-temporal (1988–2017) land surface temperature (LST) data obtained from Landsat satellite datasets	Image normalization, atmospheric correction, geometric correction	A decrease of 3–5 °C in the overall LST resulted from the urbanization of desert surfaces
Nassar et al. (2017)	Dubai, UAE	To investigate the causes of cooling in desert cities, particularly the impact of land use on the cooling effects	A time series of Landsat images, land use types, urban growth	Atmospheric correction using the Fast Line of Sight Atmospheric Analysis of Spectral Hyper cubes module within ENVI	All urban land-use types contribute in a heat sink. The largest cooling effects is generated by green areas. On the other hand, impervious surfaces are significantly responsible of the heat sink. Changes in albedo were not causally related to the urban heat sink, however, variations in urban geometry, particularly the amount of shading cast by buildings, had some influence on the magnitude of cooling
Nassar et al. (2016)	Dubai, UAE	To study the dynamics and controls of Surface Urban Heat Sinks (SUHS) and Surface Urban Heat Islands (SUHI) in desert cities	Daily imagery from the MODIS thermal sensor. Land cover and urban geometry	A Local Climate Zone (LCZ) schema was developed. Canonical correlation techniques	The daytime SUHS effect is greatest during the summer months (typically∼3.0 °C). The strongest cooling effects in open high-rise zones of the city. In contrast, the night-time SUHI effect is greatest during the winter months (typically ∼3.5 °C) with the strongest warming effects incompact mid-rise zones of the city
Radhi et al. (2013)	Manama, Bahrain	To investigate the impact of urban expansion on atmospheric urban heat islands	Weather data, master plan, land use maps, satellite images	Remote sensing, advanced statistics of weather data, GIS, numerical modelling	Air temperatures increase within the range of 2–5 °C. The magnitude of the UHI is influenced by urban activities and land use. The mean temperature is increased by 2–3 °C in new artificial islands, and by 3–5 °C in deserted urban lands with hot arid conditions
Charabi and Bakhit (2011)	Muscat, Oman	To examine the spatio-temporal variability of the canopy-level urban heat island (UHI)	Meteorological observations and mobile measurements during a span of 1 year	Spatio-temporal evolution of the UHI	The peak UHI magnitude occurs from 6 to 7 h after sunset and it is well developed in the summer season. The warm core of the UHI is located in the Highland zone of Muscat, along a narrow valley characterized by low ventilation, high business activities, multi-storied buildings and heavy road traffic
Lazzarini et al. (2013)	Abu Dhabi, UAE	to analyze the daily variation of Land Surface Temperature (LST), the derived Surface Urban Heat Island (SUHI), and the Normalized Difference Vegetation Index (NDVI) at city level	Remote sensing data from MODIS, ASTER and LANDSAT 7 sensors	Multi-sensor approach	There is an inversion of the standard SUHI phenomenon during daytime, where the downtown areas appear colder compared to the suburbs. The inversion of SUHI is accentuated mainly in the summer months with a daily difference of 5–6 K compared to 2–3 K during the winter season, while the standard SUHI can be observed during the night with values of downtown 2–3 K higher than the suburbs

## Methodology

This study uses remote sensing and GIS data to compare LSTs in eight cities in arid and semi-arid climate regions. The study was conducted using multi-spatial and multi-temporal satellite data acquired from a Landsat 5 TM (1990, 2000), and a Landsat 8 Operational Land Imager (OLI) (2018). LST was derived from Landsat 8 and Landsat 5 data. In Landsat 8, band 10 was used, while band 6 was used for Landsat 5. In this study, the images of eight cities were used, which are freely accessible from NASA and were corrected for radiometric and geometrical errors (level-1 products http://earthexplorer.usgs.gov/). The characteristics of the eight images used for the analysis are summarised in Table 3.

**Table 3:** Images used to extract information for the study area

City	Landsat Scene Identifier	Time	Mission	Resolution	Path and Row	Acquisition date	Sun Azimuth	Sensor
Kuwait	LT41650401990163XXX03	06:46:35	Landsat 4–5	30*30 M	165 × 40	12/6/1990	95.13005963	TM
	LT51650402000167RSA03	06:52:51	Landsat 5	30*30 M	165 × 40	15/6/2000	95.62793425	TM
	LC81650402018168LGN00	07:15:27	Landsat 8	30*30 M	165 × 40	17/6/2018	99.71159981	OLI/TIRS
Manamah	LT51630421990157XXX03	06:25:06	Landsat 5	30*30 M	163 × 42	6/6/1990	89.46514199	TM
	LT51630422000169RSA00	06:41:20	Landsat 5	30*30 M	163 × 42	17/6/2000	89.35602553	TM
	LC81630422018170LGN00	07:03:54	Landsat 8	30*30 M	163 × 42	19/6/2018	91.82119406	OLI/TIRS
Doha	LT51630421990157XXX03	06:25:06	Landsat 5	30*30 M	163 × 42	6/6/1990	89.46514199	TM
	LT51630422000169RSA00	06:41:20	Landsat 5	30*30 M	163 × 42	17/6/2000	89.35602553	TM
	LC81630422018170LGN00	07:03:54	Landsat 8	30*30 M	163 × 42	19/6/2018	91.82119406	OLI/TIRS
Dubai	LT51600431990168RSA00	06:06:57	Landsat 5	30*30 M	160 × 43	17/6/1990	85.22294498	TM
	LT51600432000164RSA00	06:23:05	Landsat 5	30*30 M	160 × 43	12/6/2000	86.92747462	TM
	LC81600432018181LGN00	06:45:52	Landsat 8	30*30 M	160 × 43	30/6/2018	88.54891479	OLI/TIRS
Abu Dhabi	LT51600431990168RSA00	06:06:57	Landsat 5	30*30 M	160 × 43	17/6/1990	85.22294498	TM
	LT51600432000164RSA00	06:23:05	Landsat 5	30*30 M	160 × 43	12/6/2000	86.92747462	TM
	LC81600432018181LGN00	06:45:52	Landsat 8	30*30 M	160 × 43	30/6/2018	88.54891479	OLI/TIRS
Muscat	LT51580441990154ISP00	05:55:00	Landsat 5	30*30 M	158 × 44	3/6/1990	85.02678101	TM
	LT51580442000166XXX02	06:11:09	Landsat 5	30*30 M	158 × 44	14/6/2000	83.77154935	TM
	LC81580442018167LGN00	06:33:46	Landsat 8	30*30 M	158 × 44	16/6/2018	84.64995868	OLI/TIRS
Jeddah	LT51700451990158RSA00	07:09:34	Landsat 5	30*30 M	170 × 45	7/6/1990	81.62895133	TM
	LT51700452000154XXX02	07:25:28	Landsat 5	30*30 M	170 × 45	2/6/1990	83.26328251	TM
	LC81700452018155LGN00	07:48:12	Landsat 8	30*30 M	170 × 45	4/6/2018	83.55566640	OLI/TIRS
Riyadh	LT51660431990178RSA00	06:44:02	Landsat 5	30*30 M	166 × 43	27/6/1990	85.14587724	TM
	LT51660432000174RSA02	07:00:23	Landsat 5	30*30 M	166 × 43	22/6/2000	86.20707272	TM
	LC81660442018175LGN00	07:23:18	Landsat 8	30*30 M	166 × 43	24/6/2018	84.40917787	OLI/TIRS

Spatial resolution of an imagery is important when studying the effects of the spatial pattern of land use/land cover (LULC) on LST. High temporal resolution is usually used in conjunction with extensive time coverage to investigate the air temperature UHI and to describe its temporal variation (Li et al. 2013). However, one of the shortcomings of the temporal resolution is that it fails to reflect the spatial variation of UHI. Therefore, surface temperature UHI is used to provide a simultaneous surface temperature of a large area (Li et al. 2013; Weng 2009). Different studies on UHI used remotely sensed image data with a different spatial resolution to generate different LULC maps (Vannier et al. 2011; Liu and Weng 2009; Townsend et al. 2009; Weng et al. 2004). Liu and Weng (2009), for example, used a spatial resolution of 90 m and 30 m to examine the relationship between the LST and landscape level and landscape pattern, respectively. Weng et al. (2004) found that the 120 m is the optimal resolution in explaining the relationship between LST and NDVI. Li et al. (2013) examined the importance of spatial resolution in determining the LST. They used three spatial resolutions (2.44 m, 10 m, and 30 m) to measure the spatial pattern of greenspace based on seven landscape metrics. They found that the greenspace spatial pattern is more accurately quantified using images with high spatial resolution, and hence the relationship between LST and the spatial configuration of greenspaces differ based on the spatial resolution. In this study, we used a spatial resolution of 30 m, which is the most frequently used Landsat 5 TM and Landsat 8 OLI TIRS.

Three land cover classes were identified within the study area using unsupervised classification to investigate the LST and the UHI effect as shown in Table 4. Normalized differences in the built-up index (NDBI), NDVI, and LST values were extracted from each pixel in the study area for each data point type. All the parameters introduced in this section are presented in Table 5. LST retrieval involved the following steps.

**Table 4:** Landcover classification and definitions used in this study

Classes	Definition
Urban	All built-up areas (residential, commercial, industrial, roads, parking lots, paved areas, construction sites)
Vegetation/green	All areas of natural or cultivated vegetation (parks, trees, grasses, golf courses)
Bare	All areas containing exposed and non-developed surfaces (sand, rocks, soil)

**Table 5:** Parameters utilized in this study

Parameters	Definition
*L*_λ_	Spectral radiance (W/(m^2^ sr µm))
*L*_min(λ)_	The minimum detected spectral radiance for the minimum DN
*L*_max(λ)_	The maximum detected spectral radiance for the maximum DN
*M*_L_	Radiance multiplicative scaling factor for the band (RADIANCE_MULT_BAND_n from the metadata)
*Q*_cal_	Level 1 pixel value in DN
*A*_L_	Radiance additive scaling factor for the band (RADIANCE_ADD_BAND_n from the metadata)
*T*_c_	Top of atmosphere brightness temperature, in Celsius (C)
*K*_1_	Band-specific thermal conversion constant from the metadata (K1_CONSTANT_BAND_x, where x is the thermal band number)
*K*_2_	Band-specific thermal conversion constant from the metadata (K2_CONSTANT_BAND_x, where x is the thermal band number)
*ε*	Emissivity
*P*_v_	Vegetation proportion
NDVI	Normalized difference vegetation index
NDVImin	Minimum NDVI value
NDVImax	Maximum NDVI value
TB	The at-satellite brightness temperature
*λ*	The central band wavelength of emitted radiance (= 11.45 μm) (Markham and Barker 1985)
*ρ*	*h* × *c*/*σ* (1.438 × 10^–2^ m K) with: *σ* is the Boltzmann constant (1.38 × 10^–23^ J/K), h = Planck’s constant (6.626 × 10^–34^ J s), *c *= light velocity (2.998 × 10^8^ m/s)
NIR	Near-infrared with a wavelength of 0.845–0.885 µm
R	Visible red with a wavelength of 0.630–0.680 µm
SWIR	Short wavelength infrared with a wavelength of 1.56–1.66 µm
NIR	Near-Infrared with a wavelength of 0.76–0.90 µm

Step one consisted of converting digital numbers (DN) into a spectral radiance. Images were processed into units of absolute radiance using 32-bit floating-point calculations. These values were converted to 16-bit integer values in the finished Level 1 product. The DN values ranged from 0 to 255 for Landsat 5 TM and between 0 and 65,535 for Landsat 8 OLI TIRS. They were then converted to spectral radiance values using the radiance scaling factors provided in the metadata file. To compute the spectral radiance from the DN of the thermal bands in the satellite images, the following formula was used:

1 $$L_{\lambda } = \, L_{\min (\lambda )} + \, \left[ {L_{\max (\lambda )} {-} \, L_{\min (\lambda )} } \right]{\text{DN}}/Q_{\max }$$

The values of *L*_min(*λ*)_ and *L*_max(*λ*)_ were obtained from a metadata file available in the satellite images. For Landsat 8 OLI TIRS, these values were 0.1003 and 22.0018 for *L*_min(*λ*)_ and *L*_max(*λ*)_, respectively, while for Landsat 5 TM, they were 1.2380 and 15.3030, respectively. Therefore, Eq. 1 can be simplified into the following formula:

2 $$L_{\lambda } = {\text{ ML}} \times Q_{{{\text{cal}}}} + \, A_{{\text{L}}}$$

2.1 $${\text{Landsat 8 OLI TIRS}}: \, L_{\lambda } = \, 0.1003 \, + \, 0.00033{\text{DN}}$$

2.2 $${\text{Landsat 5 TM: }}L_{\lambda } = \, 1.2380 \, + \, 0.05516{\text{DN}}$$

The spectral radiance was then converted to an at-sensor brightness temperature, which is the effective temperature that is viewed by the satellite assuming unity emissivity. The conversion formula is, therefore, as follows (Eq. 3):

3 $$T_{{\text{C}}} = \frac{{K_{2} }}{{\ln \left( {\frac{{K_{1} }}{{L_{\lambda } }} + 1} \right)}}$$

The values of *K*_1_ and *K*_2_ for Landsat 8 OLI TIRS were 774.89 W m^−2^ Sr μm and 1321.08 W m^−2^ Srμm, respectively, while they are 607.76 and 1260.56 for Landsat 5 TM, respectively. These values were obtained from the Landsat data user’s manual. The temperature is calculated in Kelvin.

The next step was to calculate the NDVI using Near-Infrared Band Imagery (NIR), the red band from the Landsat 8 and Landsat 5 images were used. NDVI is a simple graphical indicator that is commonly used to measure the greenness of land surfaces and provides a standardized method for comparing vegetation greenness between satellite images. This is based on the fact that NDVI values are positively correlated with a condition, such as vegetation canopy characteristics and the amount of green area in the pixel area of an image. The results from the NDVI formula yielded a ratio value that ranges from − 1 (which represents water) to + 1 (which represents dense green vegetation), while values around zero represent bare soil. The bands in Landsat 8 images were 5 and 4, while in Landsat 5 TH the bands were 4 and 3. Calculating NDVI is essential because, subsequently, the proportion of vegetation (*P*_v_), which is strongly correlated with NDVI, and emissivity (*ε*), which is related to the *P*_v_, must be calculated. NDVI can thus be calculated using the following equation:

4 $${\text{NDVI}} = \frac{{{\text{NIR}} - {\text{R}}}}{{{\text{NIR}} + {\text{R}}}}$$

The *P*_v_ was then calculated to get the LSE according to the following equation:

5 $$P_{{\text{v}}} = {\text{ Square }}\left( {{{\left( {{\text{NDVI }} + {\text{ NDVI}}_{\min } } \right)} \mathord{\left/ {\vphantom {{\left( {{\text{NDVI }} + {\text{ NDVI}}_{\min } } \right)} {\left( {{\text{NDVI}}_{\max } {-}{\text{ NDVI}}_{\min } } \right)}}} \right. \kern-\nulldelimiterspace} {\left( {{\text{NDVI}}_{\max } {-}{\text{ NDVI}}_{\min } } \right)}}} \right)$$

Next, the emissivity (*ℇ*) was calculated using the following equation:

6 $$\varepsilon \, = \, 0.004 \, \times P_{{\text{v}}} + 0.986$$

The value of 0.986 thus corresponds to a correction value in the equation.

The LST was corrected for spectral emissivity (*ε*), which can now be calculated (in degrees Celsius) using the at-satellite brightness temperature and land surface emissivity according to Eq. 7 (Artis and Carnahan 1982).

7 $${\text{LST}} = \left( {\frac{{T_{{\text{C}}} }}{{1 + \left( {\frac{{\lambda \cdot T_{{\text{C}}} }}{\rho }} \right) \cdot \ln \left( \varepsilon \right)}}} \right) - 273.15$$

The NDBI is calculated according to the following equations:

8 $${\text{Landsat 5: NDBI}} = \frac{{\left( {{\text{SWIR}} - {\text{NIR}}} \right)}}{{\left( {{\text{SWIR}} + {\text{NIR}}} \right)}}$$

9 $${\text{Landsat 8:NDBI}} = \frac{{\left( {{\text{NIR}} - {\text{R}}} \right)}}{{\left( {{\text{NIR}} + {\text{SWIR}}} \right)}}$$

The relationship between LST and NDBI and NDVI indices was then examined using Pearson Correlation analysis. This type of analysis was carried on in many studies related to examining the relationship between LST and urban and green areas (Li et al. 2013). We performed the statistical analysis using SPSS 23.0.

## Results and discussion

### UHIs spatial patterns and temporal variations

**Fig. 5:**
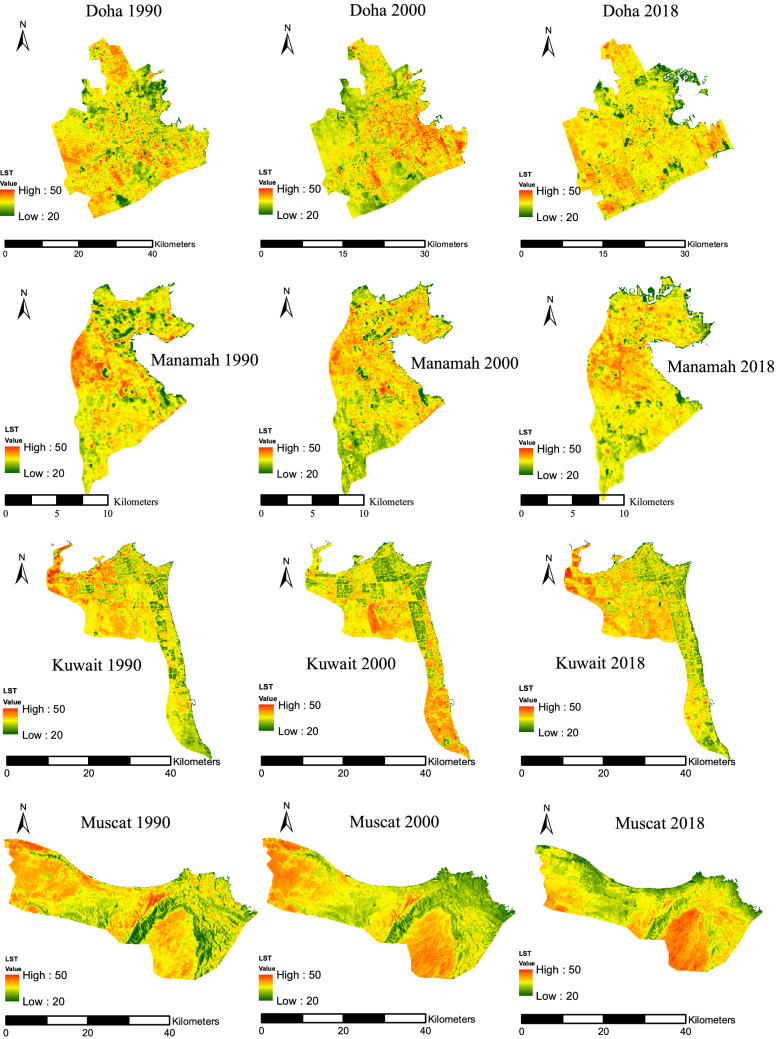

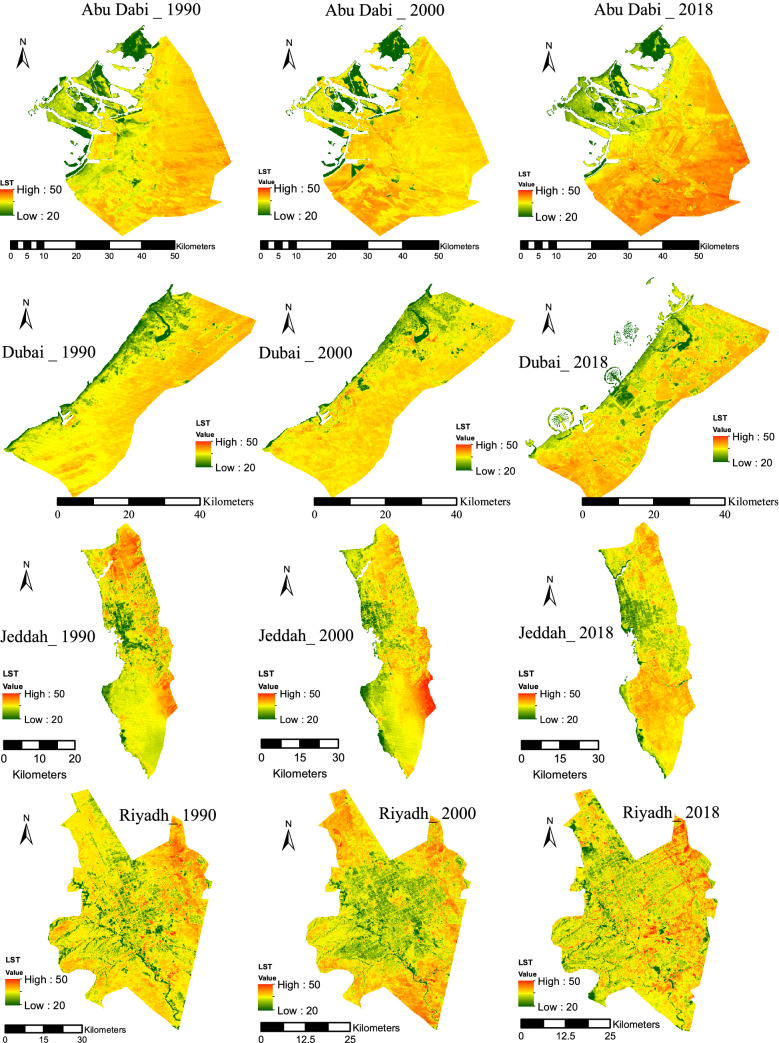
LST for the eight cities assessed in this study

The spatial LST value distributions for the different LC classes were calculated and mapped for each of the eight cities using remote sensing technology and satellite imagery. This was done to depict temperature differences, and thus the areas influenced by UHIs for the years 1990, 2000, and 2018, as shown in Fig. 5. The maps show that the surface temperatures varied between 20 and 50 °C. All cities showed similar LST spatial patterns for the three land cover types. Green areas had the lowest mean LSTs, followed by urban areas, while the bare areas had the highest mean LST values (Table 6), which highlights the influence of LC on LST values. This indicates the inversion of the UHI phenomenon in these cities, as sand constitutes the main bare surfaces, and thus absorbs more sunlight than urban or green areas due to sand’s low reflectivity. Therefore, when this factor is combined with the thermal characteristics of urban surfaces, the elevated temperature of the sandy areas can be compared to the other two types of land cover in the study area. Furthermore, this pattern shows that there is a positive correlation between bare and urban areas with mean LSTs and a negative correlation between green areas and LST.

**Table 6:** Mean surface temperatures (^o^C) for the eight cities assessed in this study

City	1990			2000			2018		
	Urban area	Green area	Bare area	Urban area	Green area	Bare area	Urban area	Green area	Bare area
Abu Dhabi	42	37	43	36	34	38	41	37	44
Dubai	42	41	44	36	34	38	40	39	42
Jeddah	29	28	31	41	40	43	36	33	37
Riyadh	39	37	40	38	34	39	44	43	44
Doha	41	40	42	37	36	37	43	41	44
Manamah	41	40	42	37	35	37	43	41	44
Kuwait	31	29	32	27	26	28	37	36	38
Muscat	44	43	45	33	30	34	36	34	37

**Fig. 6:**
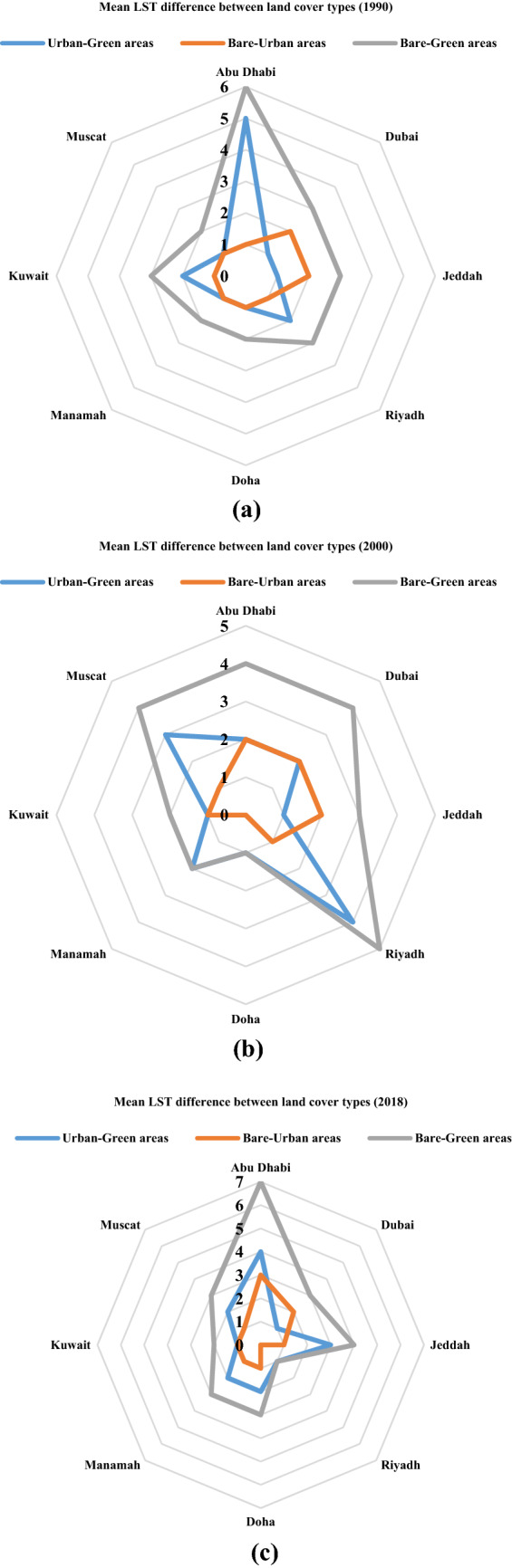
Temperature (°C) difference between different LULC in the eight cities, **a** 1990, **b** 2000, **c** 2018

The variations in the mean LST values between the three types of land cover mostly varied among the eight cities over time, as shown in Table 6. The table shows no temperature’s pattern as the values of the temperature is subject to the weather conditions at the time of capturing the image. In 1990, the difference in temperatures between the bare areas and the urban areas ranges between 1 and 2 °C, between the bare areas and green areas ranges between 2 and 6 °C, and between the urban areas and green areas ranges between 1 and 5 °C. The other years (2000 and 2018) shows a similar trend with a slightly different range in temperature between the three areas compared to 1990 period. Abu Dhabi had the highest variations in mean LST values (6 °C) between the bare areas and green areas, and a 5 °C difference between the urban areas and green areas in the year 1990, as shown in Fig. 6a. The least variability in mean LST was observed between the bare areas and green areas in Doha, Manamah, and Muscat. On the other hand, the difference in mean LST values between the bare areas and urban areas ranged between 2 °C in Dubai and Jeddah and 1 °C in the rest of the cities, in 1990. This difference was also observed for the year 2000, but the range in the mean LST between the various land covers types changed. The most considerable difference in the mean LST between the bare areas and green areas was 5 °C, and variability in the mean LST values was between 1 and 5 °C. The variations in mean LST values were less significant, as it ranged between 0 and 2 °C, as shown in Fig. 6b. On the other hand, mean LST value variations between the urban areas and green areas were between 1 and 4 °C. In 2018, the variations followed the same patterns as the previous years, as the mean LST values varied between 1 °C in Riyadh and 7 °C in Abu Dhabi, as shown in Fig. 6c. The mean LST value ranges for the bare areas and urban areas were between 0 and 3 °C, while for the urban areas and the green areas the values were between 1 and 4 °C.

### Impact of urban areas on LST in arid and semi-arid regions

The UHI spatial distributions and patterns for the urban areas are a reflection of their different components. The proximity of dense urban areas intensifies the UHI phenomena, due to human activity, the materials used for urban construction, the excessive use of artificial cooling systems, and traffic-induced air pollution (Bokaiea et al. 2016).

**Fig. 7:**
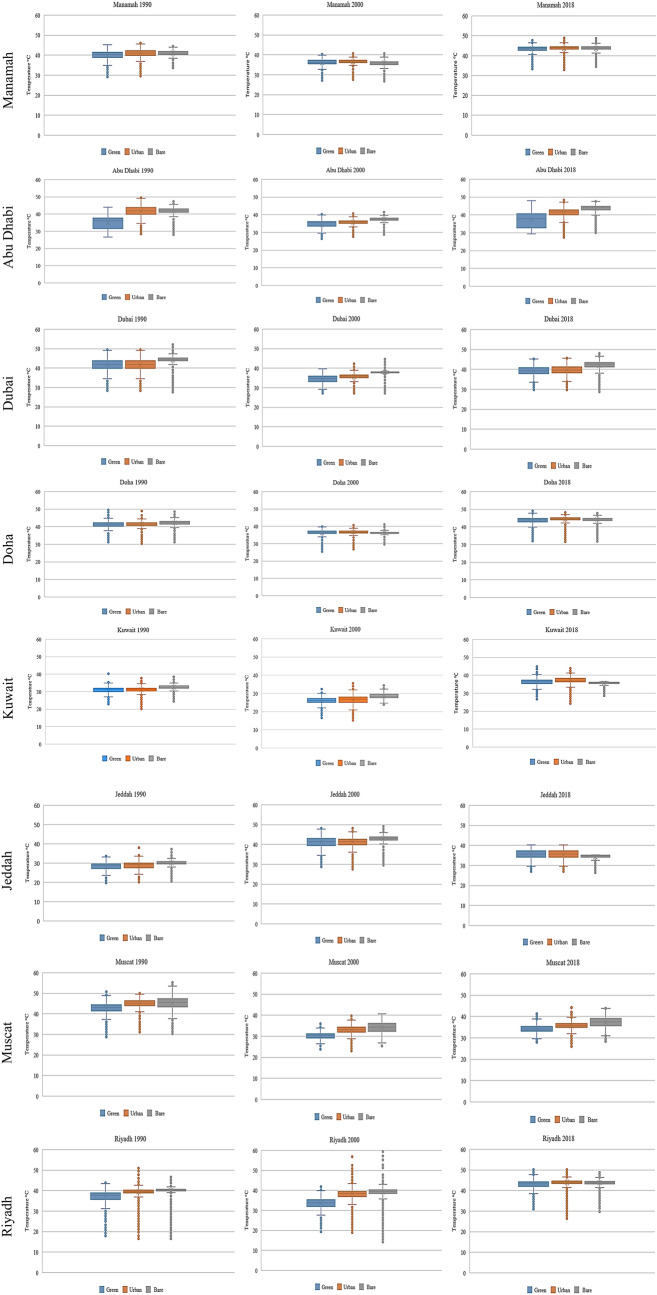
LST of the green, urban, and bare areas

The relationship between LST and the urban areas is presented in a box and whiskers plot, as shown in Fig. 7. This relationship is based on the combination of the urban area map with the LST map, which was accomplished using GIS software. Figure 7 shows that the LST of the urban areas is not homogenous but somewhat varies within each city, as there is an extensive range between the minimum and maximum temperature. This variation depends on the characteristics of the urban location. For example, as shown in Fig. 5, there are some urban areas with a higher LST than the mean LST of the bare areas. By contrast, some other urban areas have LSTs less than the mean LST of the green areas. These differences indicate that there is not one LC category that has a higher or lower LST than the others. Except for Riyadh, all the other cities are coastal, and hence the presence of sea breeze is a significant element that may significantly influence the amplitude and characteristics of the UHI effects near coastal areas.

**Fig. 8:**
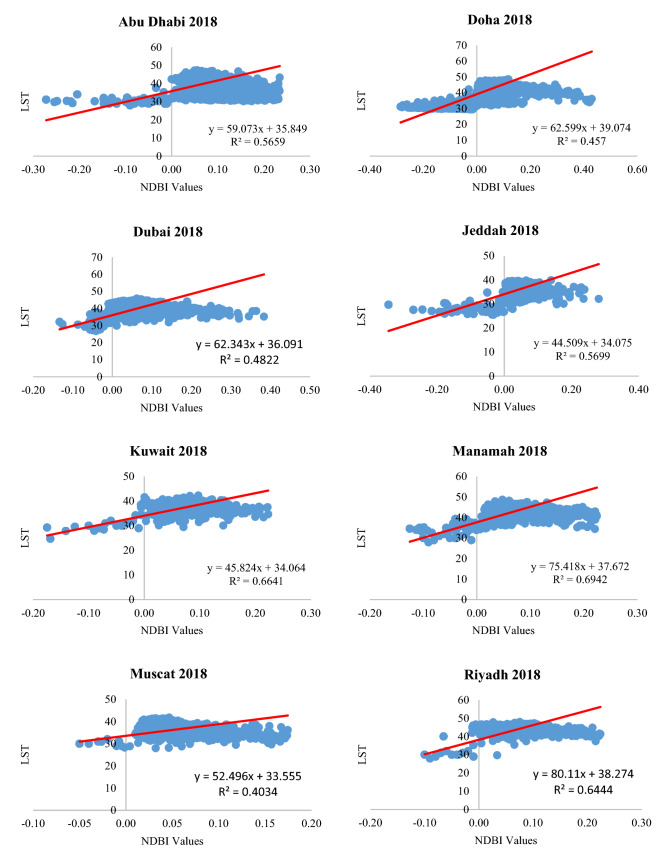
Relationship between LST and NDBI

In this study, more than 8000 points were selected using the fishnet grid tool in ArcMap 10.7 software to study the relationship between NDBI and LST in the form of two-dimensional scatter plots in the eight cities (Fig. 8). The figure shows that LST is positively correlated with the NDBI values so that LST increases by increasing the NDBI values. The NDBI values are concentrated between 0.01 and 0.2 in many of these cities with corresponding LST between 30 and 50 °C. These NDBI values represent the clustered urban areas. The negative NDBI values correspond with lower LST, as these values are urban areas associated with more green areas. Table 7 shows the statistical correlation between the LST and NDBI. The entries show that there is a significant positive correlation between LST and NDBI values in all eight cities.

**Table 7:** Correlation between LST and NDBI

City	1990			2000			2018		
	Pearson Correlation	Sig	*R*^2^	Pearson Correlation	Sig	*R*^2^	Pearson Correlation	Sig	*R*^2^
Abu Dhabi	0.683**	0.000	0.4927	0.726**	0.000	0.5347	0.752**	0.000	0.5659
Doha	0.625**	0.000	0.4281	0.684**	0.000	0.4781	0.676**	0.000	0.457
Dubai	0.501**	0.000	0.4829	0.528**	0.000	0.5146	0.546**	0.000	0.4822
Jeddah	0.677**	0.000	0.5038	0.631**	0.000	0.4692	0.755**	0.000	0.5699
Kuwait	0.763**	0.000	0.5859	0.749**	0.000	0.6104	0.803**	0.000	0.6641
Manamah	0.772**	0.000	0.5227	0.792**	0.000	0.5730	0.833**	0.000	0.6942
Muscat	0.649**	0.000	0.4372	0.683**	0.000	0.4271	0.734**	0.000	0.4034
Riyadh	0.733**	0.000	0.5106	0.772**	0.000	0.5659	0.815**	0.000	0.6444

**Correlation is significant at the 0.01 level (2-tailed)

Investigating the LST in urban areas also shows that the coastal areas have the lowest LST in the urban areas, as the sea breeze transfers cooler air to the adjacent areas of the cost and enhances wind speeds. Although LST increases as the distance from the coast increases, other factors play a significant role in increasing or decreasing LST, including the geometry of the buildings, population density, and the presence of green areas. In Riyadh, the highest LST was found in areas near the bare lands, while it decreased near the green areas. There were some locations in these cities that had intense UHIs during the study period. Investigating these places revealed that they were mainly induced by industrial and commercial land.

Urbanization also significantly increases urban temperatures. High residential, commercial, and industrial densities result in the trapping of energy in walls. This is because these materials have a high heat capacity, which leads to the excessive use of artificial cooling systems by residents, and results in anthropogenic heat in urban areas. The use of unnatural materials (such as asphalt, concrete, cement, etc.), changes the nature of these areas and increases the prevalence of dark surfaces with low albedos (greater absorption of solar energy), reduction in green areas, and increased anthropogenic heat production. Additionally, population growth influences the maximum temperatures during warm periods, resulting in elevated air temperatures in these areas and, consequently, the occurrence of UHIs (Stone et al. 2010; Liu et al. 2007). Therefore, understanding the urban fabric and the existence of LULCs in these urban areas is fundamental to creating better UHI mitigation strategies. Giannopoulou et al. (2011) investigated how urban activities contribute to the occurrence of UHIs and concluded that the formation of this phenomenon was reinforced by increased urbanization and industrialization, coupled with a decrease in green areas which resulted in increased anthropogenic heat flows.

### The impact of bare areas on LST in arid and semi-arid regions

The bare areas within and surrounding the city’s urban areas mainly consist of sand. Figure 7 shows that the bare areas have the highest LST values, and the mean LST value was the highest compared to the urban and green areas, as shown in Table 6. However, Fig. 7 shows that the LST for the bare areas varied, and there was a considerable range between the minimum and maximum temperatures for all eight cities. Furthermore, Fig. 7 shows that some bare areas in the eight cities had lower LSTs than the urban and green areas. Examining the location of the bare areas with the maximum and minimum LST revealed that the bare areas with the lowest LST were near the sea line and near green areas, and to a lesser extent, in between some of the urban areas.

One reason that the LST was high in the bare areas in these cities was due to heat concentration, sparse vegetation cover, deficient relative humidity, and topsoil aridity in the upper layers of the sandy soil. Additionally, variations in LST in the bare areas may be due to the soil properties (color, crust, type of minerals). LST was therefore affected by the water content of the topsoil in the bare areas. Sandy soils have lower water content, which results in higher LST values. Sandy soil is characterized by lower water holding capabilities and smaller thermal inertia. Therefore, during the summer, the sandy soil has lower water content and a faster depletion rate, which leads to a higher LST. Therefore, there is a positive correlation between sand and LST in these areas. On the contrary, other types of soils, such as clay soil that is used for planting, have higher water-holding capabilities. Thus, the higher the water content in the soil, the slower the depletion rate, which results in a lower LST.

### Impacts of green areas on LST in arid and semi-arid regions

**Fig. 9:**
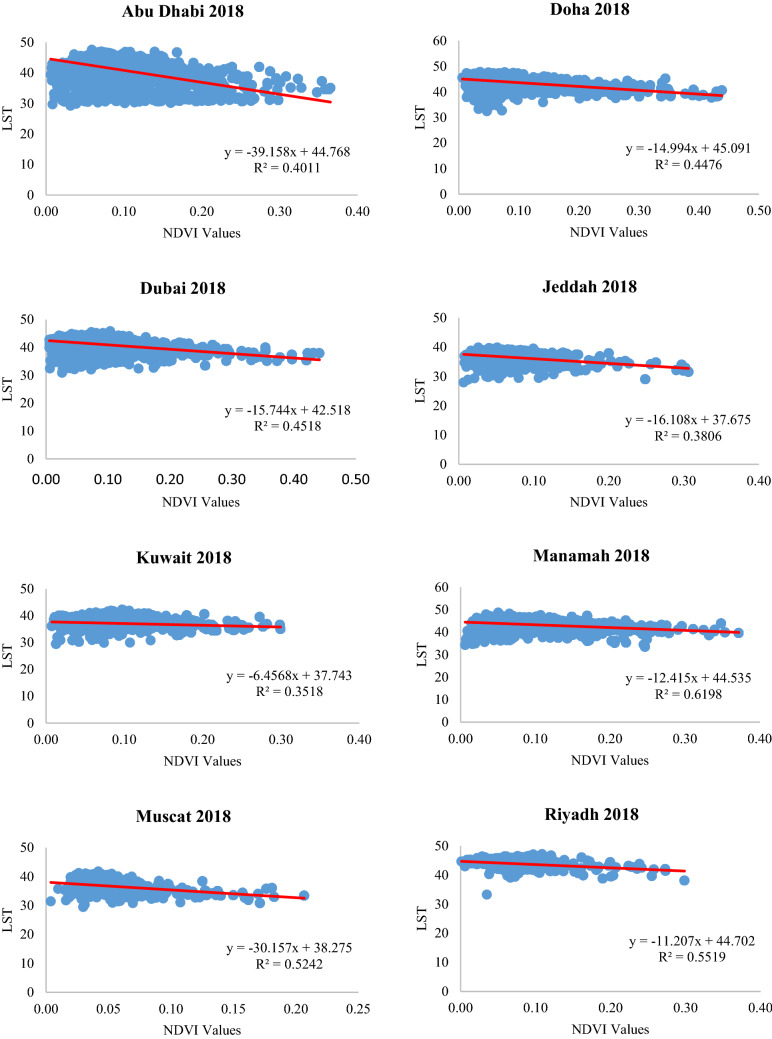
Relationship between LST and NDVI

The LST values of the green areas were derived from the map intersections of the NDVI values and the LST. More than 8000 points were selected using fishnet grid tool in ArcMap 10.7 software to study the relationship between NDVI and LST in the form of two-dimensional scatter plots in the eight cities. Figure 9 shows that there is an adverse correlation between LST and NDVI values. The LST decreases by increasing the amount of green areas. This emphasizes the role of green areas in mitigating the UHI by reducing the LST. Furthermore, the entries in Table 8 indicate that there was a siginificant negative relationship between LST and NDVI values. There was some useful information that could be extracted from this negative relationship between LST and NDVI values. First, the role of green areas is more evident in reducing the LST in the eight cities, as the areas with the highest levels of vegetation cover have the lowest LST values. This emphasizes the incontrovertible conclusion that green areas mitigate UHIs. Second, the range between the minimum and the maximum LST was high for all the cities and worth further investigation. The green areas with high temperatures represent the lower NDVI values and thus are related to sparse vegetation found in the bare areas. Although the role of green areas to reduce UHI effects is undeniable, different factors may affect the negative correlation between LST and NDVI, including the role of evapotranspiration and soil moisture. Other reasons could be the abundance, green space configuration, and the proximity of water bodies. Additionally, the dry weather, particularly in Riyadh City, plays a vital role in reducing the NDVI values because the vegetation is under water stress. The other cities were located near the sea, and hence humidity plays an essential role in elevating the NDVI values. The lowest LST for the green areas were found in areas characterized by dense vegetation and near water bodies. On the other hand, the highest LST for the green areas were related to patches of vegetation located in the bare areas. In the urban areas, the amount of vegetation has a significant effect on the distribution of the UHIs, where the size and density of the vegetation create cool island effects. Therefore, we can conclude from Fig. 7 that the influence of green areas does not always significantly reduce LST, specifically if the green areas are scattered near built-up environments and bare areas.

**Table 8:** Correlation between LST and NDVI

City	1990			2000			2018		
	Pearson Correlation	Sig	*R*^2^	Pearson Correlation	Sig	*R*^2^	Pearson Correlation	Sig	*R*^2^
Abu Dhabi	− 0.549**	0.000	0.3957	− 0.673**	0.000	0.4623	− 0.633**	0.000	0.4011
Doha	− 0.597**	0.000	0.4698	− 0.613**	0.000	0.5121	− 0.669**	0.000	0.4476
Dubai	− 0.614**	0.000	0.5169	− 0.642**	0.000	0.6235	− 0.672**	0.000	0.4518
Jeddah	− 0.579**	0.000	0.4258	− 0.573**	0.000	0.4694	− 0.617**	0.000	0.3806
Kuwait	− 0.382**	0.000	0.3714	− 0.414**	0.000	0.4103	− 0.349**	0.000	0.3518
Manamah	− 0.429**	0.000	0.5463	− 0.447**	0.000	0.5973	− 0.469**	0.000	0.6198
Muscat	− 0.586**	0.000	0.5127	− 0.618**	0.000	0.5428	− 0.665**	0.000	0.5242
Riyadh	− 0.359**	0.000	0.6192	− 0.428**	0.000	0.5854	− 0.390**	0.000	0.5519

^**^ Correlation is significant at the 0.01 level (2-tailed)

## Planning for mitigating UHI in arid and semi-arid cities

A variety of strategies, policies, and techniques had been proposed, recommended, and implemented in various cities to counterbalance the impacts of UHI effects, as well as moderate temperatures and elevate the adaptive capacities of urban areas to a warming climate. These strategies have consisted of using cooling materials, increasing the ratio of vegetation and water in the urban environment, as well as reduce anthropogenic heat, and water. However, the implementation and effectiveness of these strategies depend on many variables, and only some of these can be considered in urban planning policies or urban contexts (density, scale, design, geometry). Other elements include environmental conditions such as scale, geography, climatology, and surface topology. Therefore, this section aims to review and determine the mitigation strategies and techniques of the UHI effects in arid and semi-arid cities. The aim is to provide evidence and practical guidance for environmental professionals so that developmental policies and projects can be optimized to minimize the effects of UHIs and moderate urban microclimates in arid and semi-arid cities. Such strategies may be considered in the planning process to reduce city temperatures, particularly in the summer.

The result of this study illustrates the importance of green areas in reducing the UHI effects. Furthermore, many studies have concluded that urban greening is a robust strategy in reducing LST and hence mitigating the effects of UHIs. However, this strategy is costly in arid and semi-arid regions due to water scarcity and the resulting cost associated with it (Abulibdeh and Zaidan 2020; Abulibdeh et al. 2019b). Hence, it can be used with other strategies to reduce the negative consequences of UHIs.

Increasing the ratio of green spaces in urban areas is considered a significant way to reduce the effects of UHIs, as green spaces have substantial impacts on the thermal conditions of urban areas. Urban green areas, when combined with high sky view factors, are characterized by lower air and surface temperatures than other LULCs, such as built-up areas (Tan et al. 2015; Doick et al. 2014; Li et al. 2012). Vegetation, particularly trees, can prevent direct surface heat as a result of solar radiation by providing shade, cooling the air through generating cool island effects, by evapotranspiration and emissivity processes, and by reducing the wind speed under the canopies (Santamouris 2015; Li et al. 2012; Hamada and Ohta, 2010). Planting trees in the wind direction also elevates the cooling abilities of each tree (Tan et al. 2015; Doick et al. 2014). For example, Doick et al. (2014) concluded that the cooling effects of urban greenery were significant on warm nights with light wind speeds. Therefore, many studies suggest that creating more green areas for more efficient and stable cooling effects as the boundaries of the cooling effect may have variable effects (Myint et al. 2013; Maimaitiyiming et al. 2014; Morabito et al. 2016). Rafiee et al. (2016) investigated the role of green spaces in mitigating the effects of UHIs and found that the highest reduction extended in a 40 m radius around the greenery area.

Parks and green spaces also have a negative effect on the development of UHIs, and they reduce the energy consumption required for cooling buildings in the summer. Furthermore, green areas stabilize the temperature variations caused by the building materials. However, green areas can reduce or increase the energy consumption of adjacent buildings, depending on the size of green areas, the tree species, the climate of the site, and the ratio of trees to the surface area. Urban greening also reduces the temperature due to the evapotranspiration process and the shading of surfaces. The daily and yearly evolution of evapotranspiration thus relies on solar radiation, as this process results in the cooling of leaves and the air temperature surrounding them. Furthermore, the shade from trees can cool the atmosphere by intercepting solar radiation. This process prevents the heating of the ground surface as well as the air. Therefore, the combined effects of shading and evapotranspiration in parks and green areas result in a significant decrease in LST. This phenomenon is known as cool islands in urban areas. Furthermore, the presence of green areas in urban environments can decrease the energy consumption required for cooling in summer and reduce or stabilize the temperature changes produced by building materials. However, the cooling impact of parks and green spaces depends on the size, the type of plants, and the seasonal radiation conditions. Trees and shrubbery were found to have positive impacts on reducing LST, while grass had adverse effects on LST, and as a result, it harmed the formation of the cooling islands.

## Limitation of the study and future considerations

LSTs may differ across geographical locations and time periods. The variations in the mean LSTs between the different land cover categories may be affected by different elements, such as the time, the season of acquisition, the method used for obtaining the LSTs, the type of satellite data used, the classification of land cover categories, and the topography of the landscape. These factors, and others, need to be considered when examining and interpreting the results from different cities in the study area in future studies. This study considered investigating UHI and LST at the daytime and during the summer season. Therefore, adding nighttime data and multiple daytime readings in addition to considering the winter season could show interesting variations over time. Another interesting addition is to investigate the study area on a smaller scale to differentiate between low-density, single-story urban development with large amounts of surface asphalt and more three-dimensional built form represented by central cities or older historic areas. Furthermore, future studies can examine the large areas of bare soils within cities and examine if they generate intense localized heat islands as arid regions have many different rock types potentially with different heat absorbing characteristics as well as in many cases low, drought-tolerant vegetation. Simultaneously, future studies can investigate if the coolness of vegetated landscapes carry over onto surrounding urban areas.

Future studies on arid and semi-arid regions can consider the integrative design of cities, which is an essential concept that urban planners should consider. Integrative designs consider different thermal comfort parameters based on the existing morphologies and climates of cities. Heat in urban environments is correlated with morphology (such as urban geometry, roughness, and building density). The thermal comfort of city residents can be improved by following different strategies such as promoting good wind circulation and combining vegetation and water installations in the summer by utilizing proper land-use planning and design. Geometric features are another significant factor that influences the formation of UHIs. Tall buildings, for example, can consist of multiple surfaces that absorb and reflect solar radiation and increase the probability that heat within the cities can proliferate (Fernández et al. 2015). They also disrupt heat convection away from the city by preventing air circulation. Furthermore, the location of buildings in urban landscapes can influence the circulation of wind, as these buildings can block the wind through the urban inner spaces, which prevents cooling via convection. Therefore, future studies that focuse on examining the UHI in different cities should consider the intra-cities variation.

## Summary and conclusions

This study has analyzed the formation of the UHIs in eight different cities in arid and semi-arid regions using satelliate images Landsat-5 and Landsat-8 OLI data. The analysis is based on LC classification (urban, green, and bare areas). The results showed that the bare areas have the highest mean temperatures. However, the range between the minimum and the maximum LST in each of the land-cover categories was not always from one category. Although the mean LST in the bare areas was the highest, there were some areas in the urban and green areas with higher LST. This indicates that other factors may play a crucial role in determining LSTs and hence the UHIs. Abu Dhabi city has the highest variation in mean LST and this might be due to green cover and nearness to the water. The temperature difference between the bare areas and the urban areas ranges between 1 and 2 °C, between the bare areas and green areas ranges between 2 and 7 °C, and between the urban areas and green areas ranges between 1 and 6 °C.

Results showed that UHIs in the eight cities are function of urban surface properties, mainly built-up and green areas. In the analysis of the Pearson correlation coefficient, the LST has a significant relationship with both NDBI and NDVI indices but with different directions. The relationship between the LST and NDBI was significantly positive indicating that urban areas can elevate the UHI effect. On the other hand, the relationship between the LST and NDVI is negative indicating that the presence of the green areas mitigate the UHI effect. However, the bare areas have the highest mean LST compared to the other land cover categories. This demonstrates that urban and green areas in arid and semi-arid regions generate an overall cooling effect albite in different degrees. Although green areas are found to generated the largest cooling effect, the urban areas dominate these cities and hence are responsible for the preponderance of heat sink in these cities. Furthermore, there was spatial heterogeneity in LST across each category, depending on different variables such as the nearness to the sea or the density of the urban and green areas. Thus monitoring the time-series of LST is a useful tool to investigate and monitor the UHI growth in these cities.

A variety of strategies, policies, and techniques had been proposed, recommended, and implemented in various cities to counterbalance the impacts of UHI effects, as well as to moderate temperatures and elevate the adaptive capacities of urban areas to a warming climate. These strategies have consisted of using cooling materials, increasing the ratio of vegetation and water in the urban environment, as well as reduce anthropogenic heat and water. However, the implementation and effectiveness of these strategies depend on many variables, and only some of these can be considered in urban planning policies in arid and semi-arid cities. In such cities, it is possible to apply a variety of strategies and policies to mitigate the effects of UHIs. Based on the findings of this study, green spaces significantly influence LST variations in urban areas and hence are the essential elements to be considered in urban planning. Furthermore, the countries in the Gulf region can develop green standard initiatives and standards that require UHI mitigation actions to be applied in new buildings. These standards should represent a mandatory set of building performance standards, and utilizing high-albedo materials should be one of the recommended options for building envelops and urban infrastructure.

Future studies should consider nighttime data, multiple daytime readings, and seasonal variation to attain more depth analysis of the UHI formation in arid and semi-arid areas. Furthermore, the different effects of day-and-night as well as seasonal situation should be considered for constructing the planning recommendations.
